# The AFU Campus Climate Survey: Assessing Perceptions of Age Friendliness

**DOI:** 10.1093/geroni/igaa057.1740

**Published:** 2020-12-16

**Authors:** Lauren Bowen, Nina Silverstein, Susan Whitbourne

**Affiliations:** 1 University of Massachusetts, Boston, Boston, Massachusetts, United States; 2 University of Massachusetts Boston, Needham, Massachusetts, United States; 3 University of Massachusetts Amherst, Boston, Massachusetts, United States

## Abstract

While the AFU goals emphasize campus structures and practices (which can and should be evaluated), it is equally important to assess perceived age-friendliness by campus community members. Availability of academic and career services to students of all ages does not guarantee that older and younger students feel equally welcome and well-served. The Campus Climate Survey gathers the “perceived” data from faculty, staff, and students of all ages through a Likert-scale survey that asks perception questions about age-friendly services, facilities, and campus culture. Participants in this session will be shown how to use the CCS at their own institutions and learn its value through preliminary findings from 2,708 staff, student, and faculty responses from Massachusetts, such as: while the majority (68%) of campus staff members agreed that older adults feel welcome on campus, more than half (58%) anticipate facing age biases and challenges on campus as they age.

